# Globular C1q Receptor (gC1qR/p32/HABP1) Suppresses the Tumor-Inhibiting Role of C1q and Promotes Tumor Proliferation in 1q21-Amplified Multiple Myeloma

**DOI:** 10.3389/fimmu.2020.01292

**Published:** 2020-07-14

**Authors:** Jiadai Xu, Yifeng Sun, Jifeng Jiang, Zhao Xu, Jing Li, Tianhong Xu, Peng Liu

**Affiliations:** Department of Hematology, Zhongshan Hospital, Fudan University, Shanghai, China

**Keywords:** multiple myeloma, complement 1q, C1q receptors, globular C1q receptor, gC1qR, IGF2BP3, CKS1B

## Abstract

Immunodeficiencies are widely becoming known as important features of multiple myeloma (MM) and may promote the proliferation of malignant cells as well as confer resistance to therapy. Few studies focus on the immunomodulatory effects of the complement system on MM. This study aims to explore the role of C1q in MM patients. Plasma C1q was found to be significantly reduced in MM patients, and the amount of C1q deposited around the CD138^+^ cells in bone marrow (BM) biopsy sections was observed to be much higher, especially in the subgroup with 1q21 amplification (Amp1q21). CD138^+^ cells expressed higher levels of C1q receptors (C1qRs) than CD138^−^ cells. Patients with Amp1q21 expressed higher levels of globular C1qR (gC1qR), whereas patients without Amp21 expressed higher levels of collagen tail C1qR (cC1qR). Additionally, gC1qR was noted to suppress the MM-inhibiting role of C1q in H929, U266, and MM1S. gC1qR interacts with insulin-like growth factor 2 mRNA binding protein 3 (IGF2BP3), which also suppressed the function of C1q and regulated CDC28 protein kinase regulatory subunit 1B (CKS1B) mRNA. In summary, gC1qR suppressed the MM-inhibiting role of C1q and regulated CKS1B mRNA in promoting tumor proliferation via IGF2BP3 in 1q21-amplified MM. Our findings provide novel evidence on how MM cells evade the immune system and promote survival as well as suggest possible novel targets for future therapies of MM.

## Introduction

Multiple myeloma (MM), a hematological malignancy, is characterized by clonal plasma cells or plasma blasts that proliferate abnormally and evade death. The clinical presentation of MM mainly includes hypercalcemia, renal failure, anemia and bone destruction, and the development of severe infections. Specific cytogenetic abnormalities, including del17p, *t*_(14,16)_, *t*_(4,14)_, and 1q21 amplification (Amp1q21) (1–3), indicate the poor prognosis of MM patients. Amp1q21, which is defined as having a copy number gain (copy numbers > 2) at chromosome 1 at band q21, is a frequently encountered karyotype abnormality in MM, which is an adverse phenotype closely related to the survival of MM patients. Amp1q21 may occur in the form of isochromosomes, duplications, or jumping translocations in MM (4, 5). Despite emerging novel therapeutic strategies, response to treatment and final clinical outcomes are quite heterogeneous in MM patients. At present, this disease is considered to be mostly incurable.

It is becoming increasingly clear that immunodeficiencies serve an important role in MM and may promote the proliferation of malignant cells as well as confer resistance to therapy. To date, numerous disruptions in immune homeostasis have been reported in MM, including an immunologically hostile microenvironment and cellular immune defects (6). However, few studies actually focus on the immunomodulatory effects of the complement system on MM.

The complement system, a component of the innate immune system, has traditionally been considered to be an ancient defense mechanism that resists a broad range of invasive pathogens. However, recent evidence has prompted new perspectives on the intricate relationship between complements and tumorigenesis. Complement C1, the first component of the complement system and activator of the classical pathway, is a complex of three proteins: C1q, C1r, and C1s (7, 8). Human C1q is a collagen-like hexametric glycoprotein whose structure is similar to that of a flower, possessing six collagen-like “stalks” linked between six globular “heads” as well as a fibril-like central region (9). Usually, C1q activates the classical pathway by binding to IgG–antigen complexes. However, pertinent data have recognized that C1q possesses various independent functions associated with cancer progression that are not directly related to the complement system (10). C1q has two classical cell surface receptors (C1qRs): cC1qR binds to the collagen “stalks” tail, whereas gC1qR binds to the globular “heads” (11). Recently, numerous studies have placed emphasis on the binding of C1q to these two receptors, which have been shown to induce a number of functions related to cancer cell proliferation, survival, and progression.

To date, few studies have investigated the association between C1q and MM. In a recent retrospective study, Yang et al. suggested that C1q was markedly reduced in patients with MM, which may be considered a potential marker for tumor burden and immunodeficiency (12). However, specific reasons relating to the reduction in C1q in MM patients as well as the possible underlying mechanisms remain to be verified. This study seeks to explore the role of C1q and its involvement in the internal mechanisms of MM patients. Here, in MM patients with Amp1q21, gC1qR was found to promote MM cell line survival by suppressing the MM-inhibiting role of C1q and contributing to the stabilization of the CDC28 protein kinase regulatory subunit 1B (CKS1B) mRNA through insulin-like growth factor 2 mRNA binding protein 3 (IGF2BP3).

## Materials and Methods

### Patients and Samples

Between July 2016 and September 2018, plasma samples were obtained from five healthy donors (the NC group), 33 patients with monoclonal gammopathy of undetermined significance (MGUS), and 65 patients with newly diagnosed MM (NDMM). Bone marrow (BM) biopsies were obtained from 13 patients from the NDMM group, labeled as patients 1–6 (1q21+) and patients 1′-7′ (1q21–) in **Figure 2**. BM aspirates were obtained from an additional 41 NDMM patients, listed as patients 1″-3″ in **Figure 3A**, patients A–F in **Figure 3C**, and patients 1^(+)^-17^(+)^, and patients 1^(−)^-15^(−)^ in **Figures 3D**, **5C**, respectively.

The diagnoses for MGUS, MM, International Staging System stage, and Durie–Salmon stage were determined in accordance with the criteria of International Myeloma Working Group, 2018 (13). Electronic records of the corresponding cases were reviewed, and detailed data pertaining to the 65 NDMM plasma samples are given in Table 1. All details regarding other BM or plasma samples presented in this study may be found in Table S1.

**Table 1:** Clinical characteristics and plasma C1q levels of 65 NDMM patients.

**Clinical characteristic**	**Value**	**C1q (mg/L), median (range)**	***p*-value**
Age, median (range)	64 (43–85)		
<60, *n* (%)	23 (35.4)	168.0 (84.0–294.0)	**0.022**
≥60, *n* (%)	42 (64.6)	128.0 (4.0–272.0)	
**Sex**, ***n*** **(%)**			
Male	41 (63.0)	132.0 (4.0–294.0)	0.211
Female	24 (37.0)	159.0 (62.0–272.0)	
**DS stages**, ***n*** **(%)**			
I	15 (23.1)	183.0 (108.0–231.0)	0.173
II	7 (10.8)	126.0 (75.0–205.0)	
III	43 (66.1)	132.0 (4.0–294.0)	
**ISS stages**, ***n*** **(%)**			
I	28 (43.1)	164.5 (62.0–294.0)	0.151
II	15 (23.1)	155.0 (75.0–211.0)	
III	22 (33.8)	128.0 (4.0–219.0)	
**FISH**, ***n*** **(%)**			
del17p (+)	9/60 (15.0)	122.0 (82.0–219.0)	0.400
del13q14 (+)	32/60 (53.3)	132.5 (4.0–219.0)	0.276
1q21 amplification (+)	29/60 (48.3)	109.0 (4.0–240.0)	**0.004**
*t*_(11:14)_ (+)	12/60 (20.0)	141.0 (75.0–294.0)	0.647
*t*_(4, 14)_ (+)	6/60 (10.0)	85.5 (4.0–105.0)	**0.001**
*t*_(14, 16)_ (+)	2/61 (3.3)	95.5 (90.0–101.0)	0.226

*Plasma C1q levels were compared among different groups. One-way ANOVA with least-significant difference (LSD) or rank sum test with Kruskal–Wallis test was performed for analysis among groups. All reported p values were two-sided*.

*DS, Durie–Salmon; FISH, fluorescence in situ hybridization; NDMM, newly diagnosed multiple myeloma; ISS, International Staging System. Bold values indicate p < 0.05*.

### Immunofluorescence of Paraffin Sections

A total of 13 paraffin sections taken from BM tissue from the NDMM group stored in the pathology department of our hospital were incubated at 60°C for 2 h. Then, the slides were dewaxed in xylene I for 15 min and xylene II for another 15 min. Subsequently, the sections were immersed in absolute ethanol I, absolute ethanol II, 95% ethanol, 85% ethanol, 75% ethanol, 50% ethanol, and double-distilled water (ddH_2_O) for 5 min each. The slides were then placed in sodium citrate buffer (0.01 mmol/L, pH = 6.0) at 95°C for 15 min. After the buffer cooled completely, the slides were removed and rinsed with phosphate-buffered saline (PBS), twice each time, for 3 min. Then, 10% proteinase K was used to incubate the sections at 37°C for 15 min. After the slides were rinsed twice with PBS for 3 min each time, an adequate amount of primary antibody (anti-CD138 antibody, Thermo Fisher Scientific, Waltham, MA, USA, PA5-16918; anti-C1q antibody, Abcam, Cambridge, MA, USA, ab71940; anti-C5b-9 antibody, Novus Biologicals, Littleton, CO, USA, NBP1-05120) was added, and the slides were incubated at 37°C for 1 h. The above rinsing steps with PBS were repeated. A fluorescent secondary antibody was added to the sections and incubated at room temperature for 1 h in the dark. The BM tissues were incubated with DAPI for 2 min in the dark to visualize the nucleus (blue fluorescence). Finally, the slides were examined under an Olympus BX53 microscope (Olympus, Japan). The excitation wavelengths for green, red, and ultraviolet light were 450–480, 545–580, and 330–385 nm, respectively. The emission wavelengths for green, red, and ultraviolet light were 515, 610, and 420 nm, respectively. All immunofluorescence images were digitally captured and archived. Regions of interest (ROIs) were the green and red fluorescence surrounding the nucleus (blue). An image analysis system (Image-Pro Plus 6.0, ipwin32, American) was used to analyze all the immunofluorescence images. After the images were converted to gray scale 8 and the intensity was calibrated, the mean optical density (MOD) of ROI was measured as MOD = integrated optical density (IOD)/area (area > 200). Each sample was measured five times. The average MOD was reported to be the quantitative result of each sample.

### Collection of Mononuclear Cells and CD138^+^/CD138^−^ Cell Sorting

First, the BM specimen was centrifuged at 3,000 rpm for 10 min, and the supernatant was collected as plasma. An equal volume of PBS was added to the sedimentation and mixed well to generate solution A. Second, in a new centrifuge tube, solution A was added to the human lymphocyte separation solution (Hao Yang Biological, Tianjin, China, LTS1077), ensuring a clear interface between the two layers. After centrifugation at 3,000 rpm for 15 min, the middle white suspension layer and mononuclear cell layer were carefully pipetted into a new centrifuge tube and washed three times with PBS.

The aforementioned mononuclear cells were centrifuged at 1,000 rpm × 5 min to discard the supernatant. Then, 80 μl magnetic-activated cell separation (MACS) buffer and 20 μl of CD138-coated magnetic beads (Miltenyi Biotec, Bergisch Gladbach, Germany, MB17-R0009) were added to the cell sedimentation and mixed well. After being incubated for 15 min at 4°C, 2 ml of MACS buffer was used to wash approximately 2 × 10^7^ cells, and the tube was centrifuged at 1,000 rpm for 10 min to discard the supernatant completely. Finally, the resuspended cells were applied onto a MACS sorting column, and the cells that passed through, that is, CD138^−^ cells, were collected. Afterward, the column was removed from the separator, and the magnetically labeled cells, that is, CD138^+^ cells, were immediately flushed out three times by firmly pushing the plunger into the column with the appropriate amount of buffer. Approximately 1 × 10^7^ cells were added to 1 ml of cryopreservation solution, and the remaining cells were added to 400 μl of TRIzol (Invitrogen, Carlsbad, California, USA, 15596018). All cells were stored at −80°C.

### Flow Cytometry

To analyze the membrane gC1qR and cC1qR, cells were incubated in PBS with primary antibodies (anti-gC1qR and anti-cC1qR, Abcam, ab24733, and ab2907) after sorting at 4°C for 20 min. Then, the diluted secondary antibodies, which were anti-rabbit IgG [Alexa Fluor 647 conjugated, allophycocyanin (APC) fluorochromes, red fluorescence channel] (Cell Signaling Technology, Danvers, MA, USA, 4410S) and anti-mouse IgG [fluorescein isothiocyanate (FITC) conjugated, FITC fluorochromes, and green fluorescence channel) (Jackson Immuno Research, West Grove, PA, USA, 115-095-003), were used to stain the cells at 4°C for 20 min. Finally, the expression of the surface C1qRs was measured by flow cytometry (FC) (BD FACSCanto II, Franklin Lake, New Jersey, USA). The mean fluorescence intensity (MFI) of each fluorescence was measured using FlowJo V10 (BD FACSCanto II, USA). Each cell-based experiment was repeated at least three times.

### Interphase Fluorescence *in situ* Hybridization

The confirmation of the genetic aberration of 1q21, using a sequence-specific DNA probe for 1q21/CKS1B (Jinpujia Medical Co., Ltd., Beijing, China, F04008R-00), was analyzed by Kindstar Global Technology, China. The specific steps were done according to the protocol outlined in previous studies (14–16). Under the excitation of the red monochromatic filter, the fluorescence hybridization signal of the cells was observed with the Olympus BX51 fluorescence microscope (Olympus, Japan). The number of red spot signals was the copy numbers of chromosome 1q21. Each sample was analyzed for 200 cells, and overlapping cells were excluded.

### Cell Culture, siRNA, and Transfection

In total, three human MM cell lines (HMCLs) were selected in this study: H929, U266, and MM1S. The cells were all cultured in Roswell Park Memorial Institute (RPMI)-1640 medium (HyClone, Logan City, Utah, USA, SH30809.01). All culture media were supplemented with 10% fetal bovine serum (FBS) (Thermo Fisher Scientific, 10099141). All cells (Zhong Qiao Xin Zhou Biotechnology, Shanghai, China) were cultured in a 5% CO_2_ plus 95% O_2_ environment at 37°C.

In terms of the gC1qR, cC1qR, and IGF2BP3 knockdowns, siRNA sequences were designed and synthesized by Genomeditech, China, described in detail in Table S2. First, using a six-well plate as an example, approximately 5 × 10^5^ cells were cultured in 2 ml of antibiotic-free medium for 24 h before transfection. Then, 10 μl of 20 μM siRNA was added to 250 μl of Opti-MEM Reduced Serum Medium (Gibco, Waltham, MA, USA, 31985070), and 5 μl of Lipofectamine 3000 Reagent (Invitrogen, L3000-015) was added to another 250 μl of Opti-MEM Medium, mixed well, and incubated at room temperature for 5 min. For transfection complication, the above solutions were mixed together and incubated at room temperature for 20 min. Then, the transfection complication was added to the six-well plate, making a total volume of up to 2 ml per well. After being cultured at 37°C and 5% CO_2_ + 95% O_2_ for 48 h, mRNA levels were detected using qRT-PCR, and protein levels were detected by Western blot (WB) analysis. The experimental control group was transfected with negative control (NC) siRNA.

### RNA Preparation and Quantitative Real-Time Polymerase Chain Reaction

Total RNA was extracted by a suitable TRIzol reagent and chloroform (4:1). After 12,000 rpm × 15 min centrifugation at 4°C, the liquid was divided into three layers. The upper supernatant was moved to a new tube, and an equal volume of isopropanol was added to precipitate the RNA. After 10–50 μl of diethyl pyrocarbonate (DEPC)-treated water was added to dissolve the RNA, the RNA concentration was determined.

cDNA was synthesized according to the protocol outlined by the Revert Aid First Strand cDNA Synthesis Kit (Thermo Fisher Scientific, K1622). The expressions of gC1qR, cC1qR, IGF2BP3, and CKS1B mRNA were determined by qRT-PCR, and GAPDH was amplified to normalize the relative levels of the above mRNA. The sequences of the primers are described in detail in Table S3.

Each reaction mixture consisted of 1 μl of cDNA, 5 μl of TB Green Premix Ex Taq (Takara, Japan, RR420), 0.2 μl of ROX Reference Dye (Takara, RR420A), 0.4 μl of forward primer (5 nmol/ml), 0.4 μl of reverse primer (5 nmol/ml), and 3 μl of DEPC water, for a total volume of 10 μl. Amplification cycling was performed at 95°C for 30 s in the holding stage. In the cycling stage, 40 cycles were initially done at 95°C for 5 s and then at 60°C for 34 s. The relative mRNA expression levels were calculated using the 2^−ΔΔCT^ method.

### Western Blotting

WB was performed as previously described (17). Each experiment was repeated at least three times, with a representative experiment shown. The antibodies were as follows: anti-gC1qR antibody (Abcam, ab24733), anti-cC1qR antibody (Abcam, ab2907), anti-IGF2BP3 antibody (Abcam, ab177477), anti-β-actin antibody (Cell Signaling Technology, 4967S), and secondary horseradish-peroxidase-conjugated antibodies (Abcam, ab205719, ab205718). Five clinical drugs (bortezomib, ixazomib, lenalidomide, pomalidomide, and cyclophosphamide) were purchased from Med Chem Express, USA.

### Cell Proliferation Analysis

Cell proliferation analysis was conducted according to the manufacturer's instructions by Cell-Light EDU Apollo488In Vitro Kit (100T) (Ribo Bio, China, C10310-3) suitable for FC analysis as well as a Cell Counting Kit-8 (CCK-8) (Dojindo, Minato-ku, Tokyo, Japan, ck-04). The final concentration of recombinant human C1q (ProSpec, Rehovot, Israel, Pro-554) was 20 μg/ml. Each experiment was repeated at least three times, with a representative experiment shown.

The specific reaction of Apollo488 fluorescent dye with EDU could directly and accurately detect DNA replication activity, which is suitable for cell proliferation analysis after siRNA treatment. In view of the EDU cell proliferation assay (FITC channel, the longest excitation wavelength 490 nm, the longest emission wavelength 520 nm), a control group without EDU medium was initially set up for the dye background analysis. Second, the cell culture medium in the six-well cell culture cluster was changed to 50 μM of EDU medium and incubated for 2 h. Then, the cells were collected and centrifuged at 350 *g* for 5 min to discard the supernatant. Subsequently, the cells were incubated with 1 ml of 4% paraformaldehyde per tube for 30 min and centrifuged at 600 *g* for 10 min to discard the supernatant. The paraformaldehyde was then neutralized with 2 ml of 2 mg/ml of glycine. After being cleaned once with PBS, 500 μl of 1× Apollo staining reaction solution was added to each tube, protected from light and incubated for 10 min at room temperature, which was then centrifuged at 1,500 rpm for 5 min to discard the staining reaction solution. Finally, the cells resuspended in 500 μl of PBS were analyzed by FC. The MFI of FITC was measured by the software FlowJo V10. Mnc indicates the MFI of FITC for the negative control samples, and Mkd indicates the MFI of FITC for the knockdown samples. Inhibition ratio of proliferation (IRP) (%) = [(Mnc – Mkd)/Mnc] × 100%.

Regarding the CCK-8 assay, each experiment was run in triplicate and repeated three times. As indicated by the absorbance of the experimental well, Ab indicates the absorbance of the blank well, and Ac indicates the absorbance of the control well. Relative cell viability (%) = [(As – Ab)/(Ac – Ab)] × 100%.

### Coimmunoprecipitation Coupled With Mass Spectrometry

Total protein was obtained from HMCLs using radioimmunoprecipitation assay (RIPA) weak cell lysis buffer (Beyotime, Shanghai, China, P0013D) supplemented with protease and phosphatase inhibitor cocktails (Thermo Fisher Scientific, 87786, 78420). The samples were centrifuged at 12,000 rpm × 30 min. After the supernatant was removed to another Eppendorf tube, the total protein concentrations were measured with a bicinchoninic acid (BCA) assay (Beyotime, P0012). For the INPUT sample, 100 μl of total protein was reserved. The mouse anti-gC1qR antibody was rotated with protein A magnetic beads (Abcam, ab214286) at a ratio of 1:100 under a 4°C environment for 2 h. Then, gC1qR and its interacting proteins were immunoprecipitated with antibody conjugated magnetic beads by rotating them at 4°C overnight. After the flow through is removed, the immunoprecipitants were analyzed by mass spectrometry to explore the spectrum of the interacting proteins, and the protein database was searched to determine the protein identities. Finally, the results were further verified by WB analysis.

### RNA Binding Protein Immunoprecipitation Followed by High-Throughput Sequencing

The DEPC-treated lysis buffer, containing 150 mM of NaCl, 50 mM of Tris–HCl (pH = 7.4), 1% Triton X-100, 1 mM of ethylenediaminetetraacetic acid, 40 U/ml of ribonuclease inhibitor (TaKaRa, Kusatsu, Shiga, Japan, 2313Q), and proteinase and phosphatase cocktail inhibitors, was mixed well by agitation for 30 min. First, the rabbit anti-IGF2BP3 antibody was incubated with protein A magnetic beads pretreated with lysis buffer at a ratio of 1:100 for 2 h at 4°C with rotation. Then, the 1.5–2.0 × 10^7^ cell pellet was resuspended in 1 ml of lysis buffer after centrifugation at 1,000 rpm for 3 min. Subsequently, the samples were centrifuged at 12,000 rpm for 30 min, and the supernatant was removed to a new Eppendorf tube. For the RNA extraction of the INPUT sample, 10% of the supernatant (100 μl) was mixed with TRIzol reagent. The remaining supernatant (900 μl) was incubated with the magnetic beads–antibody complex overnight with rotation. After being washed six times with 1 ml of lysis buffer, the immunoprecipitants were directly mixed with TRIzol reagent in order to isolate the RNA as described above. All steps were performed at 2–8°C.

NEB Next Ultra Directional RNA Library Prep Kit was used as our preferred method in preparing the clusters of cDNA libraries. RNA sequencing was finally performed on Illumina Hi-Seq sequencing platforms.

### Statistical Analysis

Statistical Product and Service Solutions (SPSS) Statistics 23 (IBM, USA) was used to perform all statistical analyses. Overall survival (OS) and progression-free survival (PFS) were estimated using the Kaplan–Meier method (log-rank test). All data of *in vitro* experiments obtained from at least three separate experiments were expressed as mean ± standard deviation (SD). When continuous variables possessed equal variance, unpaired Student's *t*-tests were performed between two groups, and one-way ANOVA was used among at least three groups to determine whether an overall statistically significant difference was present. Nonparametric tests were performed when equal variances were not assumed. A two-sided *p* < 0.05 was considered to be statistically significant.

For RNA sequencing using Cutadapt (v1.9.1) (non-default parameters: – max-n 0 – minimum-length35) (18) and Trimmomatic (v0.35) (non-default parameters: SLIDINGWINDOW:4:15 LEADING:10 TRAILING:10 MINLEN:35), high-quality reads (i.e., clean reads) were acquired by removing sequencing adapters, short reads (length < 35 bp) and low-quality reads. rRNAs were removed using Bowtie 2 (v2.3.0) (19), according to the rRNA database downloaded from the National Center for Biotechnology Information in May 2019. After high-quality reads were ensured by FastQC and after being mapped to the homo genome (assembly GRCh38) using Hisat2 (v2.0.5) (non-default parameters:—rna-strandness RF—dta), the peak detection was analyzed via MACS (v2.1.2) (non-default parameters: -f BAM—nomodel-*p* 0.01). The cutoff of the *p*-value was set to 0.01. Finally, peak annotation with gene features was performed using the intersect internals function of BEDTools (v2.26.0) with default parameters.

## Results

### C1q Significantly Reduced in Multiple Myeloma Patients, Especially in the 1q21 Amplification Subgroup

**Figure 1:**
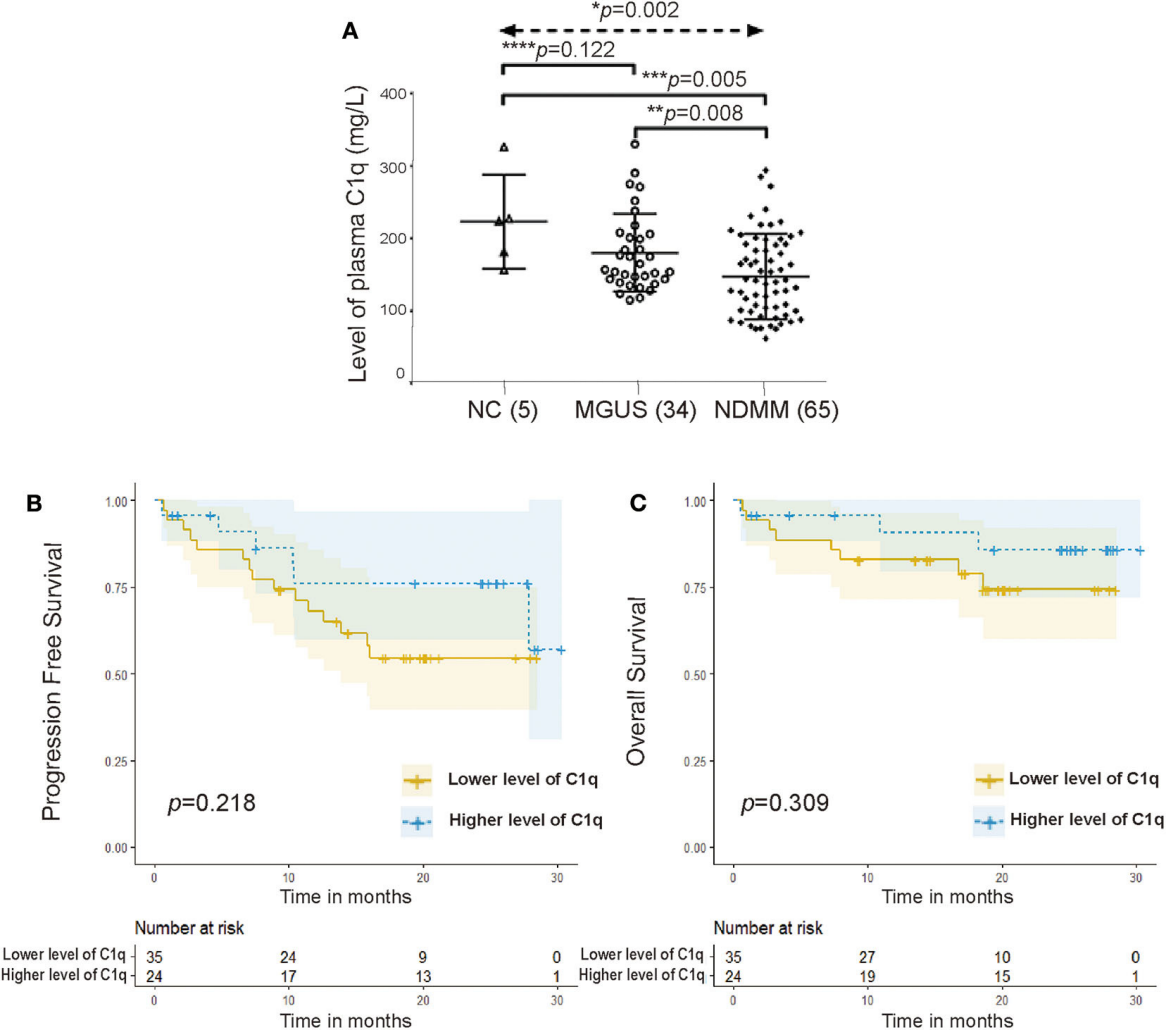
C1q significantly reduced in multiple myeloma (MM) patients. **(A)** Plasma C1q levels among the NC group (*n* = 5) (median = 224 mg/l), monoclonal gammopathy of undetermined significance (MGUS) group (*n* = 34) (median = 161 mg/l), and newly diagnosed MM (NDMM) group (*n* = 65) (median = 142 mg/l) (*p* = 0.003). Error bars represent mean ± standard deviation (SD). **(B)** Univariate survival analysis for progression-free survival (PFS) grouped by the level of plasma C1q in NDMM patients (*p* = 0.218). **(C)** Univariate survival analysis for overall survival (OS) grouped by the level of plasma C1q in NDMM patients (*p* = 0.309).

Compared with that of the NC group (*n* = 5, median = 224 mg/l, range = 157–326 mg/l) and MGUS group (*n* = 34, median = 161 mg/l, range = 115–330 mg/l), the plasma C1q level was significantly lower in the NDMM group (*n* = 65, median = 142 mg/l, range = 4–294 mg/l) (Figure 1A, *p* = 0.002). An overview of the clinical features of the 65 NDMM patients as well as the relationship with C1q levels was summarized (Table 1). In the NDMM group, patients with Amp1q21 (*p* = 0.004) had significantly lower levels of plasma C1q.

A total of 56 patients in the NDMM group received regular treatment and follow-up in our department (July 2016–September 2018). The follow-up date was up to November 15, 2019, and the median follow-up period was 19.082 months. Low levels of plasma C1q seemed to predict shorter PFS (Figure 1B, *p* = 0.218) and worse OS (Figure 1C, *p* = 0.309); however, no statistical differences were observed between the two groups.

### The Amount of C1q Deposited Around the CD138^+^ Cells in Bone Marrow Biopsy Sections Was Significantly Higher, Especially in the 1q21 Amplification Subgroup

**Figure 2:**
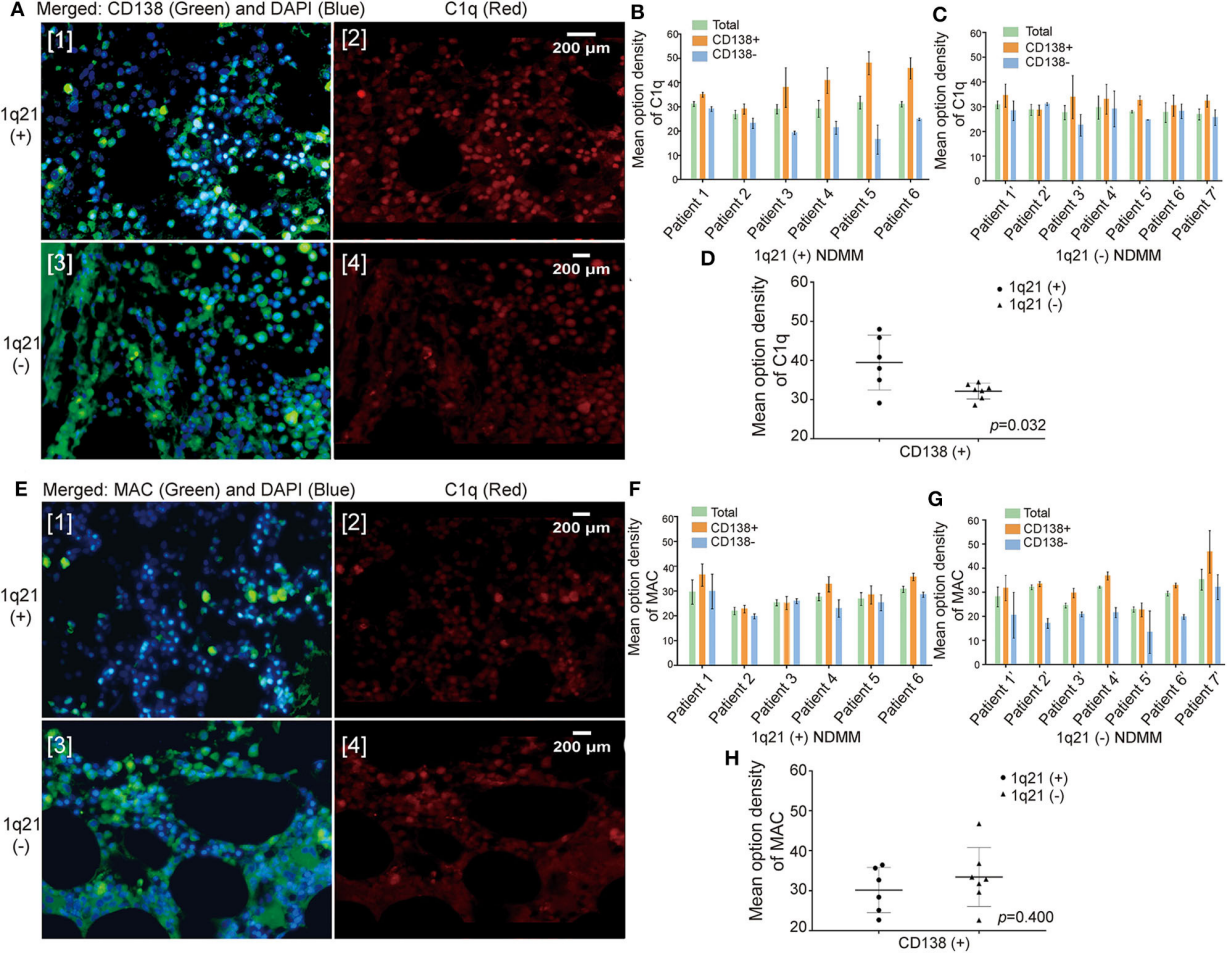
The amount of C1q deposited around the CD138^+^ cells in bone marrow (BM) biopsy sections was significantly higher, especially in the 1q21 amplification (Amp1q21) subgroup. **(A)** Double immunofluorescence labeling on BM biopsy sections: anti-CD138 antibody (green fluorescence) and anti-C1q antibody (red fluorescence). **(B)** Mean optical density (MOD) of C1q around CD138^+^ cells and CD138^−^ cells in the 1q21(+) group. Error bars represent mean ± SD. **(C)** MOD of C1q around CD138^+^ cells and CD138^−^ cells in the 1q21(–) group. Error bars represent mean ± SD. **(D)** MOD of C1q around CD138^+^ in comparison with the 1q21(+) and 1q21(–) groups (*p* = 0.032). Error bars represent mean ± SD. **(E)** Double immunofluorescence labeling on BM biopsy sections: anti-CD138 antibody (green fluorescence) and anti-membrane attack complex (anti-MAC) antibody (red fluorescence). **(F)** MOD of MAC around CD138^+^ cells and CD138^−^ cells in the 1q21(+) group. Error bars represent mean ± SD. **(G)** MOD of MAC around CD138^+^ cells and CD138^−^ cells in the 1q21(–) group. Error bars represent mean ± SD. **(H)** MOD of MAC around CD138^+^ in comparison with the 1q21(+) and 1q21(–) groups (*p* = 0.400). Error bars represent mean ± SD.

In the NDMM group, we obtained BM paraffin sections from 13 patients: six with Amp1q21 and seven without. First, we performed double immunofluorescence labeling on the BM sections using an anti-CD138 antibody (green fluorescence) and an anti-C1q antibody (red fluorescence) (Figure 2A). In all 13 NDMMs, compared with green fluorescence, red fluorescence was also relatively strong, suggesting that C1q was more likely to deposit on the membrane of CD138^+^ cells than on CD138^−^ cells (Figures 2B,C). In the 1q21(+) group (Figure 2B), the MOD of C1q around CD138^+^ cells was significantly higher than that in the 1q21(–) group (Figure 2D, *p*= 0.032), which may explain why patients in the 1q21(+) group had lower levels of plasma C1q.

To determine whether the excessive deposition of C1q around plasma cells would result in an increase in the membrane attack complex (MAC) produced following the complement-mediated activation of cell lysis, immunofluorescence double labeling was done on the BM biopsy sections using an anti-CD138 antibody (green fluorescence) as well as an anti-MAC antibody (red fluorescence) (Figure 2E). However, the results demonstrated that although the amount of MAC around CD138^+^ cells was greater than that around CD138^−^ cells, the difference was small (Figures 2F,G). Additionally, no statistically significant difference in the MOD of MAC around CD138^+^ cells was observed between the 1q21+ and 1q21– groups (Figure 2H, *p* = 0.400).

### In CD138^+^ Cells, Patients With 1q21 Amplification Expressed Higher Levels of Globular C1q Receptor, Whereas Those Without 1q21 Amplification Expressed Higher Levels of Collagen Tail C1q Receptor

**Figure 3:**
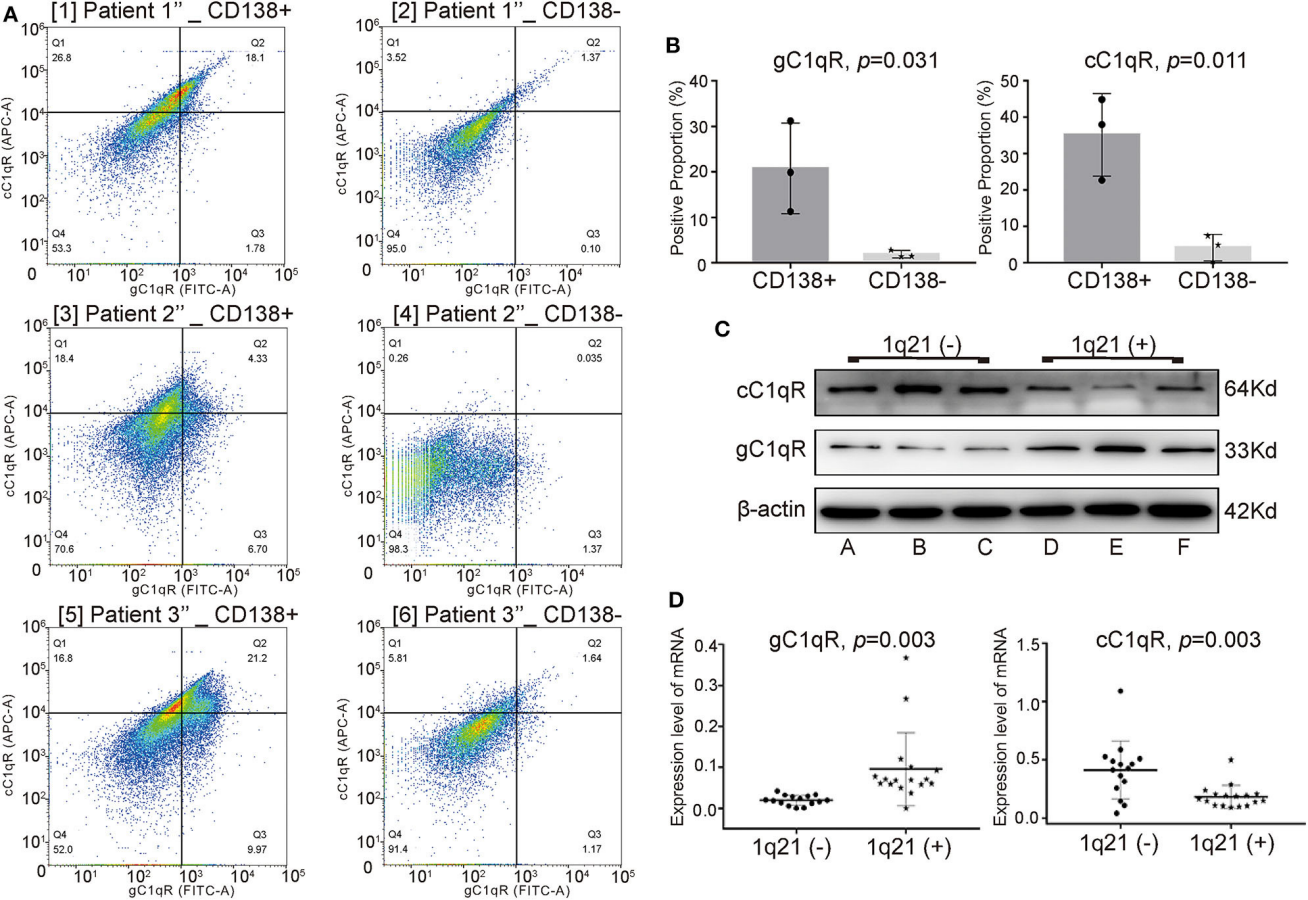
In CD138^+^ cells, patients with Amp21 expressed higher levels of globular C1q receptor (gC1qR), whereas patients without Amp21 expressed higher levels of collagen tail C1q receptor (cC1qR). **(A)** The expression levels of gC1qR and cC1qR between CD138^+^ cells and CD138^−^ cells tested by flow cytometry (FC) in three newly diagnosed multiple myeloma (NDMM) patients. **(B)** CD138^+^ cells expressed significantly higher levels of gC1qR (*p* = 0.031) and cC1qR (*p* = 0.011) than did CD138^−^ cells from the same MM patients. Error bars represent mean ± SD. **(C)** The protein levels of gC1qR and cC1qR on CD138^+^ cells between the 1q21+ group (patients A–C) and 1q21– group (patients D–F). **(D)** The mRNA levels of gC1qR (*p* = 0.003) and cC1qR (*p* = 0.003) on CD138^+^ cells between the 1q21+ group (patient 1^(+)^-17^(+)^) and 1q21– group (patient 1^(−)^-15^(−)^). Error bars represent mean ± SD.

To explore the expression of C1qRs on CD138^+^ and CD138^−^ cells, fresh BM aspirates were collected from another three NDMM patients (patients 1″-3″). Through FC, CD138^+^ cells were found to express significantly higher levels of gC1qR (Figure 3A, *p* = 0.031) and cC1qR (Figure 3B, *p* = 0.011) than did CD138^−^ cells from the same patients.

To explore whether there were differences between the expression levels of gC1qR and cC1qR among the 1q21(+) and 1q21(–) groups, BM aspirate samples were taken from another 38 NDMM patients. The protein levels of C1qRs in CD138^+^ cells from patients A–F were detected using WB analysis. Patients A–C had Amp1q21, whereas patients D–F did not have Amp1q21. The mRNA levels of C1qRs on CD138^+^ cells from patients 1^(+)^-17^(+)^ belonging to the 1q21(+) group and patients 1^(−)^-15^(−)^ belonging to the 1q21(–) group were detected via qRT-PCR.

Compared with the 1q21(–) group, the 1q21(+) group expressed significantly higher levels of total gC1qR and lower levels of total cC1qR on CD138^+^ cells at both the protein (Figure 3C) and mRNA levels (Figure 3D, *p* = 0.003 for gC1qR, *p* = 0.003 for cC1qR), suggesting that gC1qR could bind more C1q, eventually demonstrating additional C1q deposition around the plasma cells in BM biopsies from Amp1q21 patients.

### Globular C1q Receptor Suppressed the Multiple Myeloma-Inhibiting Role of C1q

**Figure 4:**
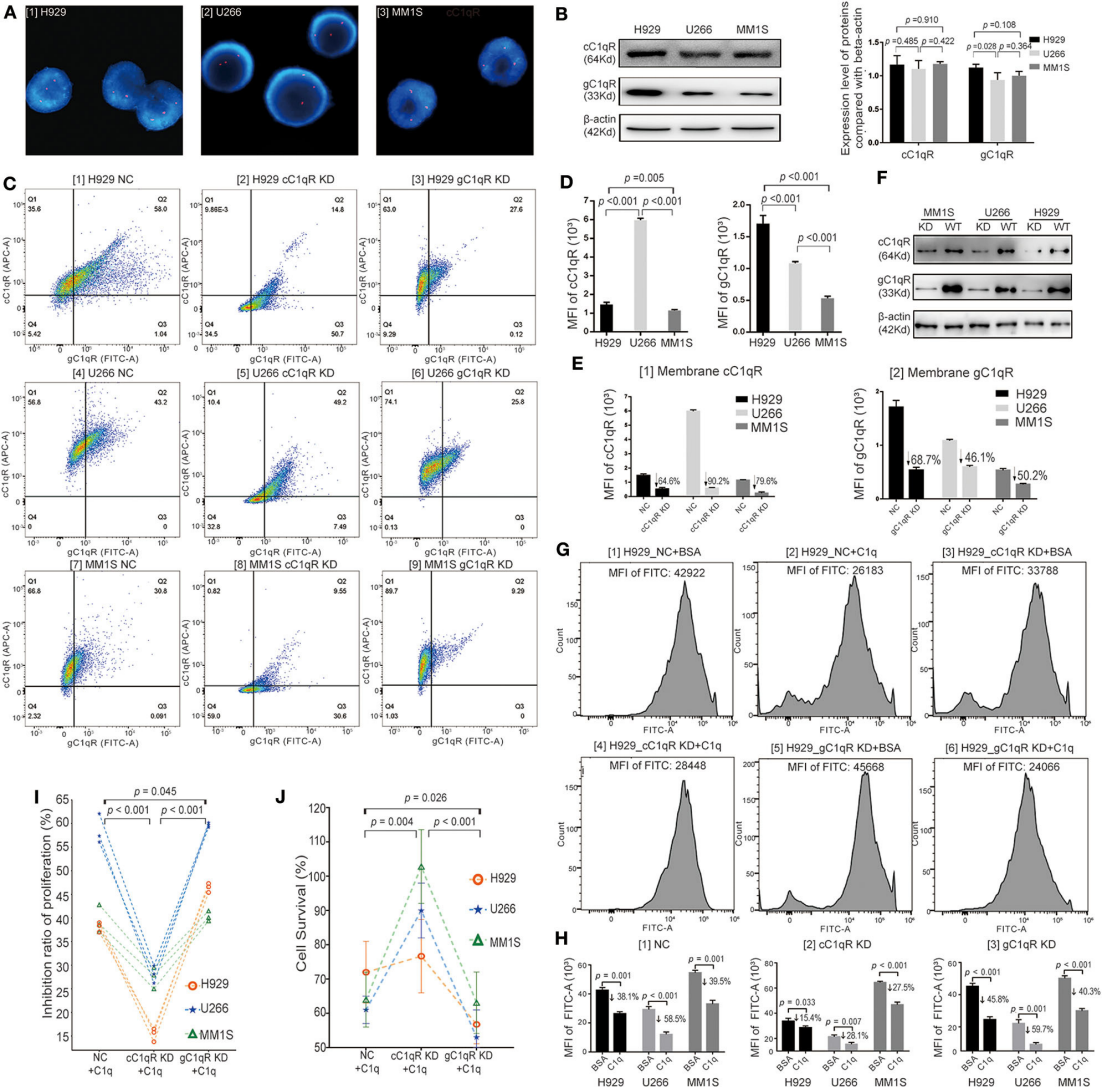
Globular C1q receptor (gC1qR) suppressed the multiple myeloma (MM)-inhibiting role of C1q. **(A)** Selected by fluorescence *in situ* hybridization (FISH) using the sequence-specific DNA probe 1q21/CKS1B, H929, U266, and MM1S having 1q21 amplification (Amp1q21) (>2 copies) were confirmed. All of them had three copies of chromosome 1q21 (three red spot signals). **(B)** The total protein expression levels of C1qRs on H929, U266, and MM1S tested by Western blot (WB). Three human MM cell lines (HMCLs) all expressed gC1qR and collagen tail C1q receptor (cC1qR), where H929 cells expressed the higher level of total gC1qR compared with U266 (*p* = 0.028) and MM1S (*p* = 0.108). **(C)** The expression levels of membrane cC1qR and membrane gC1qR on three HMCLs (H929, U266, and MM1S) analyzed by flow cytometry (FC) assay in models of NC siRNA, cC1qR KD, and gC1qR KD. **(D)** Based on the FC assay for the NC siRNA groups, the membrane protein expression levels of C1qRs on H929, U266, and MM1S were analyzed. [1] Comparison on the mean fluorescence intensity (MFI) of APC fluorescence (cC1qR) for the three HMCLs. U266 expressed the highest level of membrane cC1qR, followed by H929 and MM1S. [2] Comparison on the MFI of fluorescein isothiocyanate (FITC) fluorescence (gC1qR) for the three HMCLs. H929 expressed the significantly highest level of membrane gC1qR, followed by U266 and MM1S. **(E)** Based on the FC assay for the cC1qR KD and gC1qR KD groups, the membrane protein expression levels of knockdown C1qRs on H929, U266, and MM1S were analyzed. [1] In contrast with the NC group, membrane cC1qR was downregulated by 64.6% on H929, 90.2% on U266, and 79.6% on MM1S, on average. [2] In contrast with the NC group, membrane gC1qR was downregulated by 68.7% on H929, 46.1% on U266, and 50.2% on MM1S, on average. **(F)** Knockdown for the total expression level of gC1qR and cC1qR was verified by WB. **(G)** The EDU (FITC) assay among the NC groups, the cC1qR KD groups, and the gC1qR KD groups on H929. The value of MFI of FITC for each representative image is above each graph. **(H)** Based on the data of EDU assay, the average MFI of FITC (EDU) and mean inhibition ratios of proliferation (IRPs) on each group were measured. Error bars represent mean ± SD. [1] The average MFI of FITC (EDU) in the NC groups on each cell line. The mean IRPs of C1q against H929 (*p* = 0.001), U266 (*p* < 0.001), and MM1S (*p* = 0.001) were 38.1, 58.5, and 39.5%, respectively. [2] The average MFI of FITC (EDU) in the cC1qR KD groups on each cell line. The mean IRPs of C1q against H929 cC1qR KD (*p* = 0.033), U266 cC1qR KD (*p* = 0.007), and MM1S cC1qR KD (*p* < 0.001) were 15.4, 28.1, and 27.5%, respectively. [3] The average MFI of FITC (EDU) in the gC1qR KD groups on each cell line. The mean IRPs of C1q against H929 gC1qR KD (*p* = 0.033), U266 gC1qR KD (*p* = 0.007), and MM1S gC1qR KD (*p* < 0.001) were 45.8, 59.7, and 40.3%, respectively. **(I)** The paired-sample *t* test for IRPs of the NC group, the cC1qR KD group, and the gC1qR KD group of each cell line after incubation with C1q for 24 h at each respective time. **(J)** The cell survival probability among the NC group, the cC1qR KD group, and the gC1qR KD group on the three cell lines tested by Cell Counting Kit-8 (CCK-8). Error bars represent mean ± SD.

Three HMCLs with Amp1q21 (>2 copies) were selected by fluorescence *in situ* hybridization (FISH) using the sequence-specific DNA probe 1q21/CKS1B: H929, U266 and MM1S, all of whom had three copies of chromosome 1q21 (three red spot signals) (Figure 4A). These HMCLs all expressed gC1qR and cC1qR, where H929 cells expressed a higher level of total gC1qR than did U266 (*p* = 0.028) and MM1S (*p* = 0.108) (Figure 4B). Models of cC1qR-knockdown (cC1qR KD) and gC1qR-knockdown (gC1qR KD) among the three HMCLs were established using siRNA transfection. In order to verify the knockdown efficiency, the expression levels of membrane cC1qR and membrane gC1qR were analyzed by FC (Figure 4C). In the NC group, by comparing the MFI of APC (cC1qR), U266 was confirmed to express the highest level of membrane cC1qR, followed by H929 and MM1S (Figure 4D). By comparing the MFI of FITC (gC1qR) in the NC group, H929 was confirmed to express the significantly highest level of membrane gC1qR, followed by U266 and MM1S (Figure 4D). Membrane cC1qR was downregulated by 64.6% in H929, 90.2% in U266, and 79.6% in MM1S, on average (Figure 4E [1]). Moreover, membrane gC1qR was downregulated by 68.7% in H929, 46.1% in U266, and 50.2% in MM1S, on average (Figure 4E [2]). Knockdown in terms of the total expression level of gC1qR and cC1qR was verified by WB (Figure 4F). Here, when the membrane protein decreased, the level of total protein also decreased.

Cell proliferation analysis was first conducted by Cell-Light EDU Apollo488 *in vitro* Kit, which was suitable for the FC test. Bovine serum albumin (BSA) was used as the negative control to C1q, and the NC groups (NC + BSA and NC + C1q), cC1qR KD groups (cC1qR KD + BSA and cC1qR KD + C1q), and gC1qR KD groups (gC1qR KD + BSA and gC1qR KD + C1q) were established. After 24 h of incubation with C1q or BSA in FBS-free RPMI-1640 medium, the MFI of FITC between the NC + C1q and NC + BSA groups was compared, where the mean IRPs of C1q against H929 (*p* = 0.001), U266 (*p* < 0.001), and MM1S (*p* = 0.001) were 38.1, 58.5, and 39.5%, respectively (Figure 4H [1] NC). Accordingly, in the NC groups [Figure 4G [1][2] for H929, Figure S1A [1][2] for U266, and Figure S1B [1][2] for MM1S], C1q was observed to significantly suppress cell proliferation.

In contrast, in regard to the cC1qR KD groups [Figure 4G [3][4] for H929, Figure S1A [3][4] for U266, and Figure S1B [3][4] for MM1S], the mean IRPs of C1q against H929 cC1qR KD (*p* = 0.033), U266 cC1qR KD (*p* = 0.007), and MM1S cC1qR KD (*p* < 0.001) were still reduced; however, the degrees of reduction were slighter compared with those of the NC groups, which were 15.4, 28.1, and 27.5%, respectively [Figure 4H [2] cC1qR KD].

In terms of the gC1qR KD groups [Figure 4G [5][6] for H929, Figure S1A [5][6] for U266, and Figure S1B [5][6] for MM1S], the mean IRPs of C1q against H929 gC1qR KD (*p* < 0.001), U266 gC1qR KD (*p* = 0.001), and MM1S gC1qR KD (*p* < 0.001) were 45.8, 59.7, and 40.3%, respectively [Figure 4H [3] gC1qR KD].

In order to clearly compare the changes in cell proliferation of the NC, cC1qR KD, and gC1qR KD groups following incubation with C1q on the basis of EDU assay, Figure 4I depicts the paired-sample *t*-test for cell IRPs on the three HMCLs at each respective time. The results illustrated that the IRPs of the cC1qR KD + C1q group were significantly lower than those in the NC + C1q (*p* < 0.001) and gC1qR KD + C1q groups (*p* < 0.001). The mean IRP in the gC1qR KD + C1q group was significantly higher than that in the NC + C1q group (*p* = 0.045).

By repeating the cell treatment in the above experiment, the cell survival rate of each group of the three HMCLs was tested by CCK-8 (Figure 4J). According to the paired-sample *t* test, the average cell survival rate of the gC1qR KD + C1q group (59.2 ± 5.2%) was found to be most significantly decreased compared with that of the NC+ C1q group (63.0 ± 9.5%, *p* = 0.026) and cC1qR KD + C1q group (95.0 ± 22.6 %, *p* < 0.001). The average cell survival rate of the cC1qR KD + C1q group was significantly increased compared with that of the NC + C1q group (*p* = 0.004).

The above results suggested that C1q could inhibit the proliferation of HMCLs, although gC1qR suppressed this effect. From another perspective, in relation to the expression of gC1qR protecting HMCLs from C1q injury, it indirectly promotes the proliferation of tumor cells.

### Globular C1q Receptor Interacted With IGF2BP3 While Suppressing the Multiple Myeloma-Inhibiting Role of C1q

**Figure 5:**
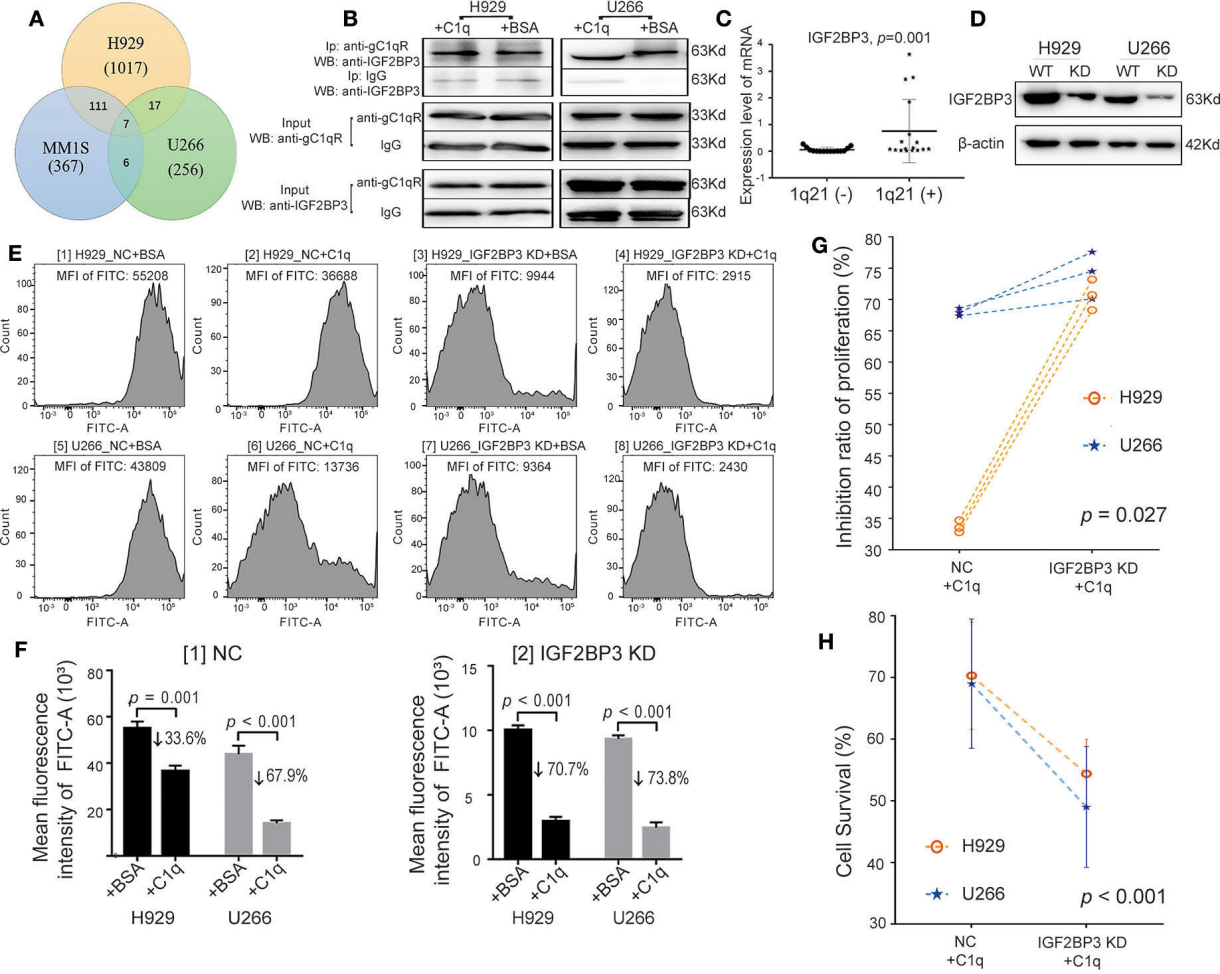
Globular C1q receptor (gC1qR) interacted with IGF2BP3, which also suppressed the multiple myeloma (MM)-inhibiting role of C1q. **(A)** Venn diagram: detection of proteins interacting with gC1qR using coimmunoprecipitation coupled with mass spectrometry (CoIP-MS). **(B)** Western blot (WB) verified that gC1qR and IGF2BP3 were indeed combined with each other. Keeping the amount of INPUT consistent, the amount of gC1qR-bound IGF2BP3 in the C1q-treated group was slightly increased compared with that in the bovine serum albumin (BSA)-treated group. **(C)** The levels of IGF2BP3 mRNA of BM CD138^+^ cells in the 1q21 (+) patient group were significantly higher than those in the 1q21 (–) group (*p* = 0.001). Error bars represent mean ± SD. **(D)** Knockdown of IGF2BP3 (IGF2BP3 KD) in H929 and U266 cells was verified by WB. **(E)** The EDU assay tested by flow cytometry (FC) [fluorescein isothiocyanate (FITC)] between the NC group and the IGF2BP3 KD in H929 and U266 cells. The value of mean fluorescence intensity (MFI) of FITC for each representative image is above each graph. **(F)** Based on the data of EDU assay, the average MFI of FITC (EDU) and mean inhibition ratios of proliferation (IRPs) on each group were measured. Error bars represent mean ± SD. [1] The average MFI of FITC (EDU) in the NC group on each cell line. The mean IRPs of C1q for H929 and U266 was 33.6 and 67.9%, separately [2] The average MFI of FITC (EDU) in the IGF2BP3 KD group on each cell line. The mean IRPs of C1q for H929 IGF2BP3 KD and U266 IGF2BP3 KD were 70.7 and 73.8%, respectively. **(G)** Based on the data of EDU assay, with the paired-sample *t* test for IRPs of C1q on H929 and U266 cells at each respective time (*p* = 0.027). **(H)** The cell survival among the NC group and the IGF2BP3 KD in H929 and U266 cells by Cell Counting Kit-8 (CCK-8) (*p* < 0.001). Error bars represent mean ± SD.

Through coimmunoprecipitation coupled with mass spectrometry (CoIP-MS), of the 1,017 types of proteins identified in the H929 group, 256 in the U266 group, and 367 in the MM1S group, a total of seven proteins were demonstrated in the overlapping regions, which interacted with gC1qR (Figure 5A, Table S4). Among these proteins, IGF2BP3 has been reported to act as a critical regulatory factor in certain cancers. Therefore, the data were further verified using WB analysis. As MM1S expressed the lowest level of gC1qR, H929, and U266 were selected for further investigation. The results demonstrated that, while keeping the amount of INPUT consistent, the amount of gC1qR-bound IGF2BP3 in the C1q-treated group was slightly increased compared with that in the BSA-treated group (Figure 5B). Additionally, qRT-PCR was used to detect BM CD138^+^ cells from the above 38 NDMM patients. Moreover, compared with the 1q21(–) group [*n* = 15, patients 1^(−)^-15^(−)^], IGF2BP3 mRNA expression levels were found to be significantly increased in the 1q21(+) group [*n* = 17, patient 1(+)−17(+)] (Figure 5C, *p* = 0.001).

By performing siRNA transfection, the models regarding IGF2BP3 knockdown (IGF2BP3 KD) were established (Figure 5D). Furthermore, with the use of BSA as the negative control to C1q, the NC group (NC + BSA and NC + C1q) and the IGF2BP3 KD group (IGF2BP3 KD + BSA and IGF2BP3 KD + C1q) were created. In regard to the EDU assay (Figure 5E), in the IGF2BP3 KD group, the mean IRPs of C1q on each cell line were 70.7% for H929 and 73.8% for U266 (Figure 5F [2]), which were significantly higher than those in the NC group (33.6% for H929, 67.9% for U266) (Figure 5F [1]). Figure 5G depicts the paired-sample *t* test for IRPs of C1q on the two HMCLs at each respective time.

After the cell treatment was repeated, the cell survival rate of each group of H929 and U266 was analyzed by CCK-8. The mean cell survival rate of the IGF2BP3 KD + C1q group (51.7 ± 7.2%) was found to be significantly lower than that in the NC + C1q group (69.65 ± 8.7%) (Figure 5H, *p* < 0.001).

### IGF2BP3 Regulated and Interacted With CKS1B mRNA

**Figure 6:**
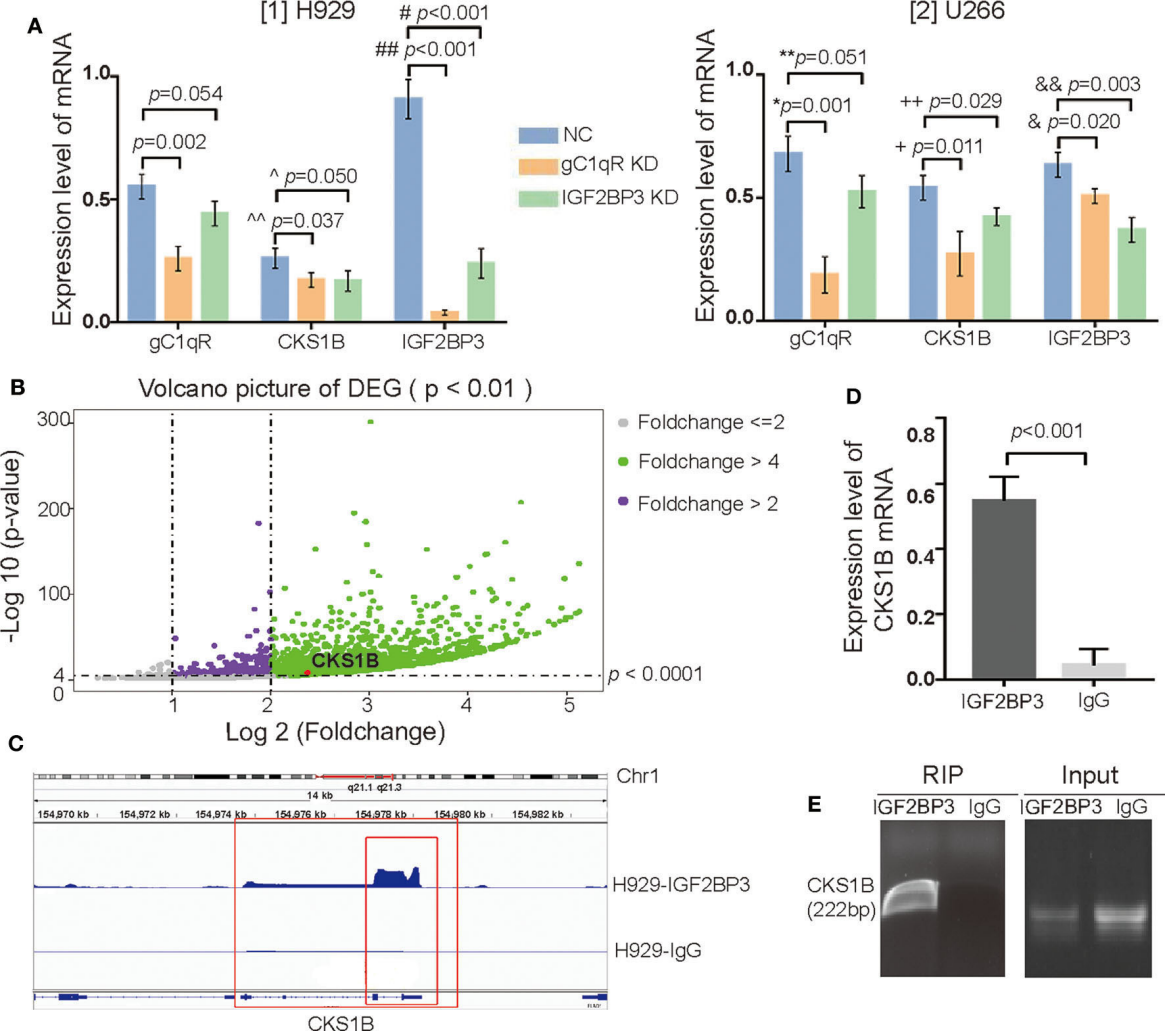
IGF2BP3 not only regulated but also interacted with CKS1B mRNA. **(A)** The mRNA levels of gC1qR, IGF2BP3, and CKS1B in the groups of NC, gC1qR KD, and IGF2BP3 KD were detected by qRT-PCR in H929 and U266. Error bars represent mean ± SD. **(B)** Volcano picture of differentially expressed genes (DEGs): enrichment analysis showed a total of 8,733 targets in the H929 cell line for the libraries from RIP. **(C)** Through integrative genomics viewer, CKS1B mRNA located at chromosome 1q21.1 to 21.3 was confirmed as one of the targets of the IGF2BP3 protein. **(D)** IGF2BP3 protein could interact with CKS1B mRNA, which was verified by qRT-PCR. Error bars represent mean ± SD. **(E)** IGF2BP3 could interact with CKS1B mRNA, which was verified by agarose gel electrophoresis.

As observed in the qRT-PCR, when gC1qR or IGF2BP3 was downregulated, the expression level of CKS1B mRNA was significantly decreased in both H929 (Figure 6A [1], ^^p = 0.037 for gC1qR KD, *p* = 0.050 for IGF2BP3 KD) and U266 (Figure 6A [2], +*p* = 0.011 for gC1qR KD, ++*p* = 0.029 for IGF2BP3 KD). Similarly, when gC1qR was downregulated, the expression level of IGF2BP3 mRNA was significantly decreased (Figure 6A [1] for H929, ##*p* < 0.001, Figure 6A [2] for U266, &*p* = 0.020). However, when IGF2BP3 was downregulated, the expression level of gC1qR mRNA was not significantly different in H929 [Figure 6A [1], *p* = 0.054] and U266 [Figure 6A [2], ^**^p = 0.051].

To isolate IGF2BP3-bound mRNAs, RIP-seq using an anti-IGF2BP3 antibody in H929 was performed. In regard to RIP libraries, the result obtained an average of 26.6 million reads. By subtracting the mRNAs that interacted with isotype IgG, the enrichment analysis demonstrated a total of 8,733 targets of IGF2BP3 proteins in H929 in the volcano picture for differentially expressed genes (DEGs). Interestingly, with the use of an integrative genomics viewer, CKS1B mRNA located at chromosome 1q21.1 to 21.3 was observed to be a target (Figures 6B,C). Furthermore, after verification with qRT-PCR (Figure 6D, *p* < 0.001) and agarose gel electrophoresis (Figure 6E), IGF2BP3 protein was confirmed to interact with CKS1B mRNA.

### Effect of Clinical Drugs on Globular C1q Receptor and IGF2BP3

**Figure 7:**
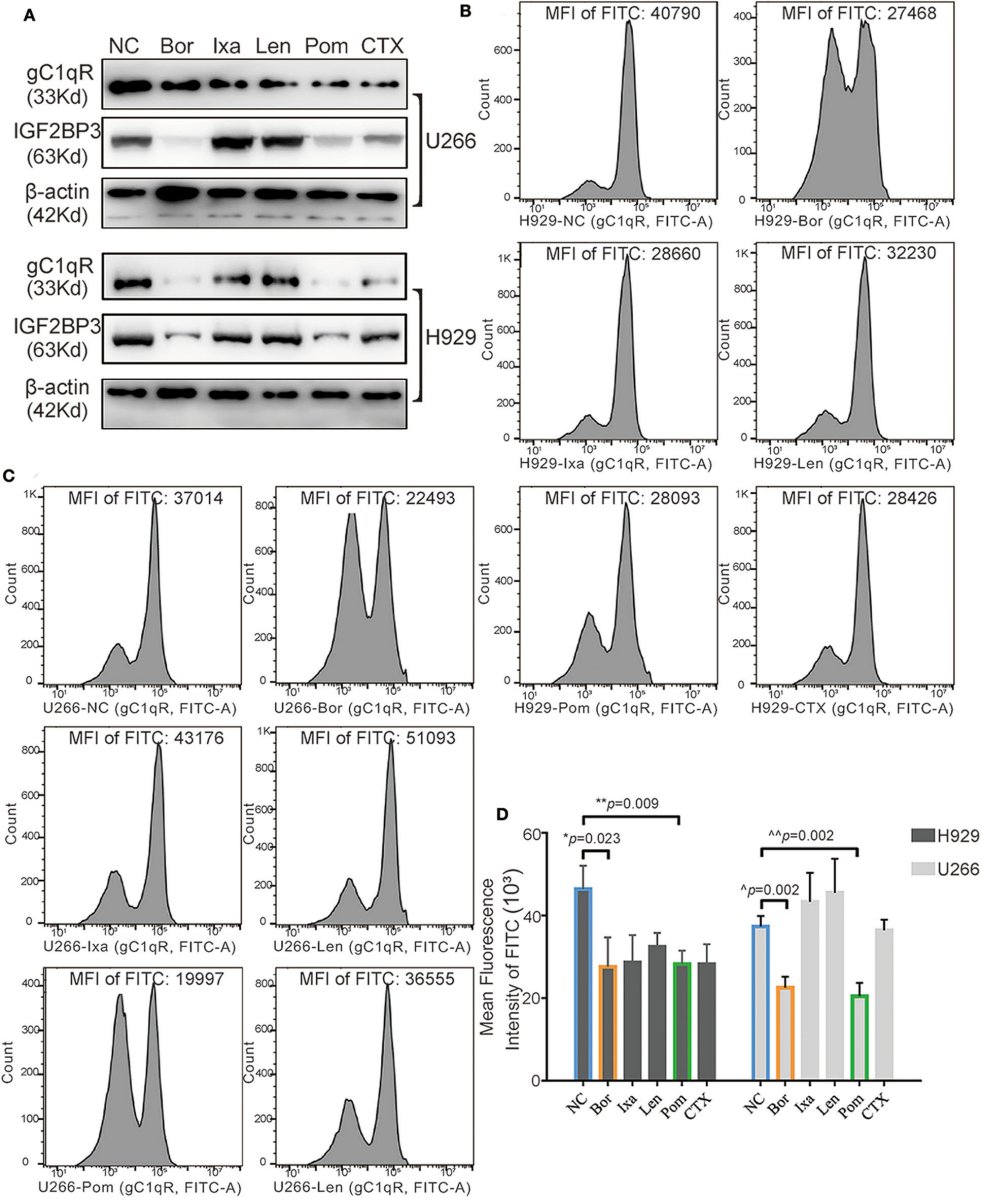
Effect of the five clinical drugs on globular C1q receptor (gC1qR) and IGF2BP3. **(A)** The gC1qR and IGF2BP3 protein expression levels of gC1qR and IGF2BP3 after adding five clinical drugs to H929 and U266 for 24-h incubation tested by Western blot (WB). **(B)** The gC1qR protein expression levels after adding five clinical drugs separately to H929 for 24-h incubation tested by flow cytometry (FC) [fluorescein isothiocyanate (FITC) fluorescence)]. The value of mean fluorescence intensity (MFI) of FITC for each representative image is above each graph. **(C)** The gC1qR protein expression levels after adding five clinical drugs separately to U266 for 24-h incubation tested by FC (FITC fluorescence). The value of MFI of FITC for each representative image is above each graph. **(D)** Average MFI of FITC fluorescence (gC1qR) after adding five clinical drugs separately to H929 and U266 for 24-h incubation tested by FC. Error bars represent mean ± SD.

**Figure 8:**
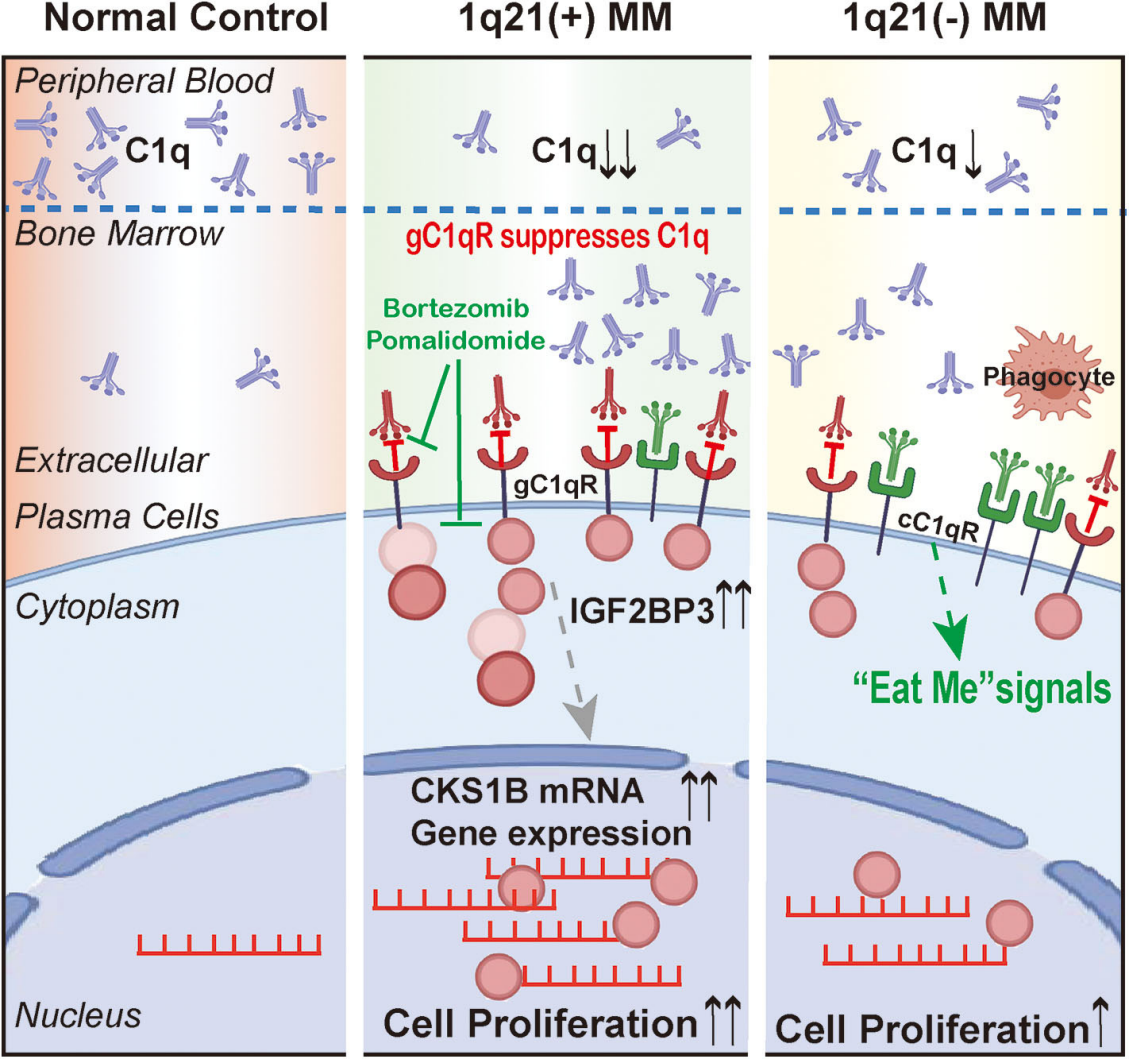
The schematic figure to illustrate the findings and conclusion of this whole study.

Five commonly used drugs in the treatment of MM patients were selected, which contained two types of proteasome inhibitors, that is, Bor and Ixa; two kinds of immunomodulatory drugs, that is, Len and Pom; and CTX. According to the IC50 concentrations, the drugs were separately added to H929 and U266 and incubated for 24 h. WB showed that (Figure 7A), Bor, Pom, and CTX significantly inhibited the expression of gC1qR and IGF2BP3 simultaneously. In contrast, FC showed that Bor and Pom still performed well in H929 (Figures 7B,D, *p* = 0.023 for Bor, *p* = 0.009 for Pom) and U266 (Figures 7C,D, *p* = 0.002 for Bor, *p* = 0.002 for Pom). Overall, Bor and Pom were found to perform very well in suppressing the expression of gC1qR and IGF2BP3. Finally, a schematic figure (Figure 8) was used to illustrate the findings and conclusion of this whole study.

## Discussion

This study provides novel evidence that gC1qR can suppress the tumor-inhibiting role of C1q and regulate CKS1B mRNA through IGF2BP3 in MM patients with Amp1q21. Thus, far, research has clearly demonstrated that immune evasion, angiogenesis, and metastasis are potentially important in tumor survival and progression. Prior studies have noted that complements regulate tumor growth and metastasis through their deposition in tumor tissues in a variety of cancers (20, 21). As the first element of the complement system, C1q was found to take part in the regulation of cellular events, modulation of cell differentiation, suppression of autoimmunity, and clearance of cell debris, which do not necessarily involve complement activation (22, 23).

Different theories exist regarding C1q's independent role in tumor growth. Bulla R et al. demonstrated that C1q acted as a cancer-promoting factor in five kinds of invasive malignant neoplasms, including colon cancer, melanoma, adenocarcinomas in lung, and breast and pancreatic cancers (10). However, other studies have demonstrated that C1q plays a potentially protective role in certain cancers. Kaur et al. found that C1q could induce apoptosis in an ovarian cancer cell line (24). Hong et al. reported that by activating the tumor suppressor WWOX, C1q also induced apoptosis in prostate cancer cells (25). Additionally, Silvio Bandini et al. confirmed that in mammary cancer cells, C1q suppresses tumor angiogenesis and induces apoptosis. Through a bioinformatics analysis, Alessandro Mangogna et al. showed the dual role that C1q plays. They highlighted that high levels of C1q had a favorable prognostic role in basal-like breast cancer for disease-free survival as well as in the OS of HER2-positive breast cancer for OS. However, in lung adenocarcinoma and clear cell renal cell carcinoma, C1q performed a pro-tumorigenic role (26). However, little is known on whether C1q plays a direct or indirect role in regulating MM growth. Yang et al. suggested that in patients with MM, C1q was markedly reduced at diagnosis and recovered to normal levels at remission. C1q appears to be a potential marker for MM burden as well as immunodeficiencies (12). In accordance with literature, C1q was reduced significantly in MM patients compared with NC and MGUS in this study. A low level of plasma C1q served as an adverse factor for OS and PFS, although the *p* > 0.05, which were possibly due to the relatively small sample size. Interestingly, the level of C1q was far lower in patients with Amp1q21. Further analysis showed that C1q was largely deposited around CD138^+^ cells in BM biopsy sections, especially in patients with Amp1q21. However, no evidence was found regarding the excessive deposition of C1q in MM patients with Amp1q21 influencing the amount of MAC deposited in BM sections produced after the complement-mediated activation of cell lysis. These phenomena may be due to C1q independently participating in the regulation of MM cell proliferation.

In plasma cell disorders, chromosomal aberrations are common. According to numerous investigations, almost 50% of MM patients possess Amp1q21 (27, 28). Ichiro Hanamura et al. performed a comprehensive analysis to evaluate a large cohort of NDMM and confirmed that Amp1q21 was an independent poor prognostic factor. Additionally, Amp1q21 indicated a shortened postrelapse survival in patients as well as a higher risk in the transition of SMM to active NDMM (28). According to Abramova et al. the 5-years OS rate in patients with Amp1q21 was 43.5%, nearly half than that in patients without Amp1q21 (79.4%, *p* = 0.07) (29). Recently, Amp1q21 patients were further divided into two subgroups: the group with three copies and the group with at least four copies. The difference variation was low in the overall 5-years OS and PFS between the two subgroups; however, postrelapse survival was significantly reduced in at least four copies of the 1q21 group. However, whether Amp1q21 is a cause or consequence of poor prognosis as well as aggressive progression remains unclear. The molecular and cellular mechanisms of Amp1q21 and its related genes require further elucidation.

To our knowledge, this study is the first to report on the specific mechanisms of interaction between C1q, C1qRs, and MM patients with Amp1q21. Prior studies have suggested that by binding to a wide range of cellular surface molecules, C1q could function broadly and participate in several physiological and pathological processes. More notably, the expression of C1qRs was found to be upregulated in many types of tumor cells (11). In the present study, two of the best-known C1qRs (8, 30), namely, cC1qR and gC1qR, had significantly different expression levels between the 1q21+ and 1q21– groups. The data showed that compared with the 1q21– group, the 1q21+ group expressed significantly higher levels of gC1qR and lower levels of cC1qR in CD138^+^ cells, indicating that these two receptors may play opposing roles. Previous studies have shown that cells expressing surface cC1qR, a prophagocytic signal, are more sensitive to apoptosis when binding with C1q, whereas cells deficient in cC1qR are relatively insensitive to apoptotic stimuli (31). The data from previous studies collectively confirmed that increased gC1qR expression exists in epithelial breast tumors as well as lung, prostate, liver, and colon cancers (11, 32, 33). Additional studies have further demonstrated that gC1qR promotes carcinogenesis and tumor progression (34). Niu et al. and Jiang et al. both demonstrated that elevated expression of gC1qR increased breast cancer risk and was correlated with poor survival in breast cancer patients (33, 35). We reviewed studies in the last 10 years relating to the correlation of gC1qR expression with PFS and OS in patients with different tumor types, and we summarized representative results in Table 2 (36–41). However, no studies have investigated the role of C1qRs in MM in-depth. The results of this study suggested that in the three MM cell lines with Amp21, gC1qR played a suppressive role in the inhibition of MM proliferation by C1q. Essentially, gC1qR contributed to MM growth, which aligned with the results that analyzed other types of tumors. Accordingly, it is inferred that the overexpression of gC1qR in malignant cells serves as a reason for the poor prognosis of patients with Amp1q21.

**Table 2:** Review of the studies relating to the correlation of gC1qR expression with PFS and OS in patients with different tumor types.

**Country (year)**	**Patients (number)**	**gC1qR overexpression vs. lower expression**		**Reference**
		**PFS or DFS**	**OS**	
China (2013)	Stage III–IV primary ovarian carcinoma (*n* = 131)	Shorter PFS (median PFS: 30.36 vs. 63.6 months, *p* < 0.001)	Shorter OS (median OS: 35.28 months vs. >50% survival at the last 60 months of follow up, *p* < 0.001)	(36)
China (2015)	Endometrial cancer (*n* = 188)	Shorter DFS (*p* = 0.022)	Shorter OS (*p* = 0.025)	(39)
China (2015)	Breast cancer (*n* = 233)	NA	Shorter OS (*p =* 0.03)	(35)
USA (2016)	Pancreatic cancer (*n* = 34)	Increasing in soluble gC1qR levels were noted with disease progression (*p* = 0.005)	NA	(41)
China (2016)	Gastric cancer (*n* = 181)	NA	Shorter OS [*p =* 0.004, HR (95% CI) = 1.730 (1.187–2.522)]	(38)
China (2019)	Pancreatic ductal adenocarcinoma (*n* = 89)	NA	Similar OS between patients with high and normal level of nucleus gC1qR (*p* = 0.312); Shorter OS in patients with higher cytoplasm gC1qR (*p* < 0.001).	(40)
USA (2019)	Malignant pleural mesothelioma (*n* = 265)	NA	Better OS among patients receiving neoadjuvant chemotherapy (NAC) (median 25 vs. 11 months; *p* = 0.020); better OS among patients without NAC (No-NAC) but who received postoperative chemotherapy (median OS 38 vs. 19 months; *p* = 0.0007)	(37)

*PFS, progression-free survival; DFS, disease free survival; OS, overall survival; NA, not available; HR, hazard ratio; 95% CI, 95% confidence interval; gC1qR, globular C1q receptor*.

One unanticipated finding in our study was that the gC1qR in MM cell lines with Amp21 interacted with IGF2BP3, and the amount of combined IGF2BP3 did not decrease when gC1qR bound to C1q. Current research on the IGF2BP family suggests that IGF2BP3 is undoubtedly an oncofetal protein that plays an essential role in cancers from onset. Abundant evidence has clarified that the expression of IGF2BP3 could enhance the invasive potential of malignant cells and correlate with poor outcomes and progressive metastases in human cancers (42–44). The results of this study indicate that, in contrast to the 1q21– MM group, patients with 1q21+ expressed significantly higher levels of IGF2BP3. Furthermore, IGF2BP3 was also shown to suppress C1q so as to ensure prolonged survival and knockdown IGF2BP3, which significantly inhibited the proliferation of MM cell lines. The above results indicated that the inhibition of C1q may result from the combination of gC1qR and IGF2BP3, and the cancer-promoting role of IGF2BP3 is reflected in that of previous studies.

The CKS1B gene is located at the smallest amplified region between 153 and 154 Mb on chromosome 1q21. Owing to chromosome Amp1q21, the CKS1B expression is increased. In this study, the downregulation of either gC1qR or IGF2BP3 could reduce the expression CKS1B mRNA. Moreover, gC1qR could regulate the expression of IGF2BP3. Finally, it was somewhat surprising to observe that IGF2BP3 interacted with CKS1B mRNA. Therefore, we hypothesized that IGF2BP3 may stabilize CKS1B mRNA by binding to this molecule, influencing the cell cycle. These findings, although preliminary, may help foster understanding regarding the cohort of MM patients with Amp1q21, interaction between the microenvironment and interior molecular mechanisms.

Finally, regarding whether a newer generation of MM drugs may affect the analyzed MM-promoting proteins, the effects of Bor and Pom on gC1qR and IGF2BP3 were observed to be most significant. As we did not specifically design further experiments to address this result, a literature review was conducted. Kay Reen Ting et al. showed that compared with treatment responders, the level of C1q in the nonresponse group was higher. On the basis of on the nano high-performance liquid chromatography (HPLC) and mass spectrometry analyses, the corresponding authors developed a panel that included C1q in order to predict the responses of MM patients to treatment, including Bor (45). Accordingly, a close connection between C1q and Bor was established in their study. Liu XL et al. showed that Chinese MM patients with Amp1q21 responded well to a Bor-based regimen; however, obtaining long-term benefits proved difficult (46). According to the STRING database, IGF2BP3 mainly participates in functional interaction networks with CD44, HMGA2, LIN28A, IGF2BP1, and ELAVL2. Alessandro Canella et al. put forward that the downregulation of CD44 is mediated by miR-9-5p, targeting IGF2BP3, which could directly strengthen CD44 mRNA stability by binding to it. Furthermore, Chad C. Bjorklund et al. demonstrated that CD44 mediates resistance to Len in MM (47), but Len was found to not function well.

The insights gained from this study may provide an understanding on the regulation of the microenvironment to the interior of MM cells from the perspective of the complement system. Various limitations existed in this study, however. First, the relatively small sample size of MM patients may generate potential bias in the interpretation of the results. Further studies with increased sample sizes could provide more definitive evidence. Second, because the limited BM samples were stored at −80°C for a relatively long time, the expression levels of the membrane cC1qR and membrane gC1qR on CD138^+^ cells between 1q21(+) patients and 1q21(–) patients could not accurately be tested by FC or WB. Therefore, through *in vitro* cell experiments, the total level of cC1qR or gC1qR was confirmed to be significantly less while the amount of membrane protein was correspondingly reduced. Third, in order to develop an overall understanding of the relationship between C1q, C1qRs, and MM, a genuine mouse model of MM is required to further establish the roles of C1q, gC1qR, and IGF2BP3. Fourth, we did not explore the deeper underlying mechanisms concerning CKS1B mRNA regulation via IGF2BP3 and whether other molecules are involved. Finally, this study questioned why drugs belonging to the same type have the same pharmacological mechanisms, such as Bor and Ixa, and Len and Pom, which have completely different effects on gC1qR and IGF2BP3 and may be further explored in our next study.

In summary, the evidence collected from MM patients revealed that plasma C1q was significantly reduced, which may be due to additional C1q deposition in the BM microenvironment. These phenomena were more significant in the Amp1q21 group, and the MM-inhibiting C1q-mediated effect was partially eliminated by binding with gC1qR, which interacted with IGF2BP3. Additionally, this study introduced a novel functional role for IGF2BP3 in regulating CKS1B mRNA. Our findings provide new perspectives in how MM cells evade the immune system and promote survival as well as suggesting possible novel targets for the future treatment of MM.

## Data Availability Statement

The clinical data used to support the findings of this study are restricted by the ethics committee in Zhongshan Hospital in order to protect patients' privacy. The datasets used and/or analyzed during the current study are available from the corresponding author (Peng Liu, liu.peng@zs-378hospital.sh.cn) on reasonable request.

## Ethics Statement

The studies involving human participants were reviewed and approved by the Ethics Committee of Fudan University, Zhongshan Hospital. The patients/participants provided their written informed consent to participate in this study.

## Author Contributions

JX designed the study, performed the experiments, and wrote the original draft. YS performed parts of the experiments and did the analysis. JJ and ZX visualized the data. JL and TX collected the clinical data. PL supervised the experiments, edited the draft, and acquired the research funding. All authors contributed to the article and approved the submitted version.

## Conflict of Interest

The authors declare that the research was conducted in the absence of any commercial or financial relationships that could be construed as a potential conflict of interest.
