# Supplementary material for: Globular C1q Receptor (gC1qR/p32/HABP1) Suppresses the Tumor-Inhibiting Role of C1q and Promotes Tumor Proliferation in 1q21-Amplified Multiple Myeloma
Source: Front Immunol. 2020 Jul 14;11:1292. doi: 10.3389/fimmu.2020.01292 (PMC7372013; doi:10.3389/fimmu.2020.01292)
Supplement: Supplementary file 1 [file Table_1.DOCX]

Supplementary Material

## Supplementary Table S1

| **Table S1. Basic clinical characteristics of patients/health donors contributing BM/Plasma samples** | | | | | | | | | |
| --- | --- | --- | --- | --- | --- | --- | --- | --- | --- |
| Characteristic | NC | MGUS | NDMM | | | | | | |
| Samples type | Plasma | Plasma | BM biopsies | | BM aspirates (CD138+/CD138- cells) | | | | |
| Experiments | Elisa | Elisa | IF-P | | FC | WB | | RT-PCR | |
| Location | Fig 1A | Fig 1A | Fig 2 | | Fig 3A | Fig 3C | | Fig 3D,5C | |
| Group (1q21: (+) or (-)) | NC | MGUS | (+) | (-) | (+): (-) =  2: 1 | (+) | (-) | (+) | (-) |
| Number of cases | 5 | 34 | 6 | 7 | 3 | 3 | 3 | 17 | 15 |
| Age | | | | | | | | | |
| Median | 30 | 62.5 | 68.5 | 60 | 67 | 64 | 57 | 65 | 60 |
| Range | 28-31 | 35-86 | 56-75 | 52-71 | 67-69 | 58-74 | 50-64 | 43-81 | 46-73 |
| <60, n (%) | 5(100.0) | 13(38.2) | 1(16.7) | 2(28.6) | 0 (0.0) | 1 (33.3) | 2 (66.7) | 7 (41.2) | 7 (46.7) |
| ≥60, n (%) | 0 (0.0) | 21 (61.8) | 5 (83.3) | 5 (71.4) | 3(100.0) | 2 (66.7) | 1 (33.3) | 10(58.8) | 8 (53.3) |
| Sex, n (%) | | | | | | | | | |
| Male | 3 (60.0) | 27 (79.4) | 3 (50.0) | 4 (57.1) | 2 (66.7) | 1 (66.7) | 1 (66.7) | 9 (52.9) | 13(86.7) |
| Female | 2 (40.0) | 7 (20.6) | 3 (50.0) | 3 (42.9) | 1 (33.3) | 2 (33.3) | 2 (33.3) | 8 (47.1) | 2 (13.3) |
| DS* Stages, n (%) | | | | | | | | | |
| Ⅰ | / | / | 1 (16.7) | 2 (28.6) | 0 (0.0) | 0 (0.0) | 0 (0.0) | 1 (5.9) | 4 (26.7) |
| Ⅱ | / | / | 0 (0.0) | 0 (0.0) | 0 (0.0) | 0 (0.0) | 0 (0.0) | 3 (17.6) | 1 (6.7) |
| Ⅲ | / | / | 5 (83.3) | 5 (71.4) | 3 (100.0) | 3(100.0) | 3(100.0) | 13 (76.5) | 10 (66.7) |
| ISS Stages, n (%) | | | | | | | | | |
| Ⅰ | / | / | 4 (66.7) | 5 (71.4) | 1 (33.3) | 1 (33.3) | 2 (66.7) | 6 (35.3) | 7 (46.7) |
| Ⅱ | / | / | 1 (16.7) | 1 (14.3) | 1 (33.3) | 0 (0.0) | 1 (33.3) | 3 (17.6) | 4 (26.7) |
| Ⅲ | / | / | 1 (16.7) | 1 (14.3) | 1 (33.3) | 2 (66.7) | 0 (0.0) | 8 (47.1) | 4 (26.7) |
| *DS: Durie-Salmon. | | | | | | | | | |

## Supplementary Table S2

| **Table S2. The siRNA sequences for gC1qR, cC1qR and IGF2BP3 knockdown** | |
| --- | --- |
| siRNA | Sequence (5'-3') |
| gC1qR（h）-si-1 | GCACCAGGAGUACAUUACUUU |
|  | AAAGUAAUGUACUCCUGGUGC |
| gC1qR（h）-si-2 | UCUGAAUGGAAGGAUACUAAU |
|  | AUUAGUAUCCUUCCAUUCAGA |
| gC1qR（h）-si-3 | CCUUGGACUGGGCCUUAUAUG |
|  | CAUAUAAGGCCCAGUCCAAGG |
| cC1qR(h）-si-1 | UGGUGCAGUUCACGGUGAAAC |
|  | GUUUCACCGUGAACUGCACCA |
| cC1qR(h）-si-2 | CUCCCGAUCCCAGUAUCUAUG |
|  | CAUAGAUACUGGGAUCGGGAG |
| cC1qR(h）-si-3 | CGUCUACUUCAAGGAGCAGUU |
|  | AACUGCUCCUUGAAGUAGACG |
| IGF2BP3（h）-si-1 | CGGUGAAUGAACUUCAGAAUU |
|  | AAUUCUGAAGUUCAUUCACCG |
| IGF2BP3（h）-si-2 | GCAGGAAUUGACGCUGUAUAA |
|  | UUAUACAGCGUCAAUUCCUGC |
| IGF2BP3（h）-si-3 | UCUGCGGCUUGUAAGUCUAUU |
|  | AAUAGACUUACAAGCCGCAGA |
|  |  |

## Supplementary Table S3

| **Table S3. The sequences of primers.** | |
| --- | --- |
| **Primer** | **Sequence (5'-3')** |
| gC1qR-forward | AACAACAGCATCCCACCAAC |
| gC1qR-reverse | AGCCTCGTCTTCTTGTCCAA |
| cC1qR-forward | CCCCAGTGATTCAGAACCCT |
| cC1qR-reverse | GCCAAACTCCTCAGCGTATG |
| IGF2BP3-forward | TGGAAAAGGAGGCAAAACGG |
| IGF2BP3-reverse | CTTCTGTTGTTGGTGCTGCT-3 |
| CKS1B-forward | CTGTTGGGAGTTGCTTGGAG |
| CKS1B-reverse | CCATCCCTGACTCTGCTGAA |
| GAPDH-forward | GAAGGTGAAGGTCGGAGTC |
| GAPDH- reverse | GAAGATGGTGATGGGATTTC |
|  |  |

## Supplementary Table S4

| **Table S4. Proteins interacting with gC1qR in 3 HMCLs.** | |
| --- | --- |
| **Gene names** | **Protein names** |
| IGF2BP3 | Insulin-like growth factor 2 mRNA-binding protein 3 |
| KRT1 | Keratin 1 |
| HRNR | Hornerin |
| SLC25A11 | Mitochondrial 2-oxoglutarate/malate carrier protein |
| RPS18 | 40S ribosomal protein S18 |
| RPS24 | 40S ribosomal protein S24 |
| RPS25 | 40S ribosomal protein S25 |
