# Supplementary material for: Globular C1q Receptor (gC1qR/p32/HABP1) Suppresses the Tumor-Inhibiting Role of C1q and Promotes Tumor Proliferation in 1q21-Amplified Multiple Myeloma
Source: Front Immunol. 2020 Jul 14;11:1292. doi: 10.3389/fimmu.2020.01292 (PMC7372013; doi:10.3389/fimmu.2020.01292)

Supplementary Material

Supplementary Figure S1

Figure S1. Results of EDU assay in the NC groups, the cC1qR KD groups and the gC1qR KD groups for U266 (A) and MM1S (B).

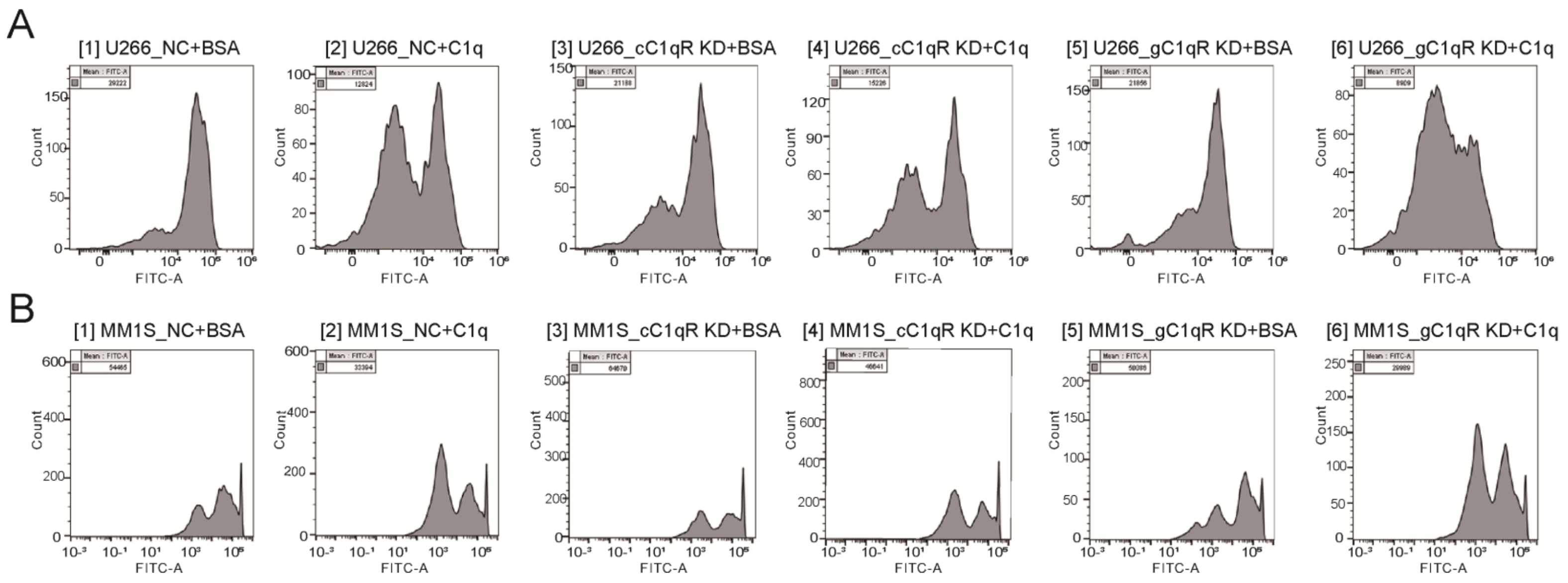

Supplement: Supplementary file 2 [file Data_Sheet_1.PDF]
